# Adenosine Transporter ENT4 Is a Direct Target of EWS/WT1 Translocation Product and Is Highly Expressed in Desmoplastic Small Round Cell Tumor

**DOI:** 10.1371/journal.pone.0002353

**Published:** 2008-06-04

**Authors:** Hongjie Li, Gromoslaw A. Smolen, Lisa F. Beers, Li Xia, William Gerald, Joanne Wang, Daniel A. Haber, Sean Bong Lee

**Affiliations:** 1 Genetics of Development and Disease Branch, National Institute of Diabetes & Digestive & Kidney Diseases, National Institutes of Health, Bethesda, Maryland, United States of America; 2 Massachusetts General Hospital Cancer Center, Harvard Medical School, Charlestown, Massachusetts, United States of America; 3 Department of Pharmaceutics, University of Washington, Seattle, Washington, United States of America; 4 Department of Pathology, Memorial Sloan-Kettering Cancer Center, New York, New York, United States of America; University of Hong Kong, China

## Abstract

**Background:**

Desmoplastic Small Round Cell Tumor (DSRCT) is a highly aggressive malignancy that affects mainly adolescents and young adults. A defining characteristic of DSRCT is a specific chromosomal translocation, t(11;22)(p13;q12), that fuses *EWS* with *WT1*, leading to a production of two isoforms of chimeric transcription factor, EWS/WT1(−KTS) and EWS/WT1(+KTS). The chimeric proteins are thought to play critical roles in various stages of oncogenesis through aberrant transcription of different genes, but only a few of these genes have been identified.

**Methodology/Principal Findings:**

We report the identification of a new target of EWS/WT1, *ENT4* (equilibrative nucleoside transporter 4) which encodes a pH-dependent adenosine transporter. *ENT4* is transcriptionally activated by both isoforms of EWS/WT1 as evidenced by promoter-reporter and chromatin immunoprecipitation (ChIP) analyses. Furthermore, *ENT4* is highly and specifically expressed in primary tumors of DSRCT as well as in a DSRCT cell line, JN-DSRCT-1. Treatment of JN-DSRCT-1 cells with adenosine analogs, such as 2-chloro-2′-deoxyadenosine (2-CdA), resulted in an increased cytotoxic response in dose- and pH-dependent manner.

**Conclusions/Significance:**

Our detailed analyses of a novel target of EWS/WT1 in DSRCT reveal an insight into the oncogenic mechanism of EWS-fusion chromosomal translocation gene products and provide a new marker for DSRCT. Furthermore, identification of *ENT4* as a highly expressed transcript in DSRCT may represent an attractive pathway for targeting chemotherapeutic drugs into DSRCT.

## Introduction

Desmoplastic small round cell tumor (DSRCT) is a rare and poorly understood neoplasm with extremely poor prognosis. Tumors are frequently found in the serosal surface of abdomen and pelvis, although tumors arising from other sites have been described [1]–[3]. It is characterized histologically by solid nests of small neoplastic cells surrounded by dense stromal components consisting of fibroblasts and hyperplastic blood vessels (reviewed in [4]). The tumor cells are positive for epithelial, mesenchymal, and neural markers, thereby confounding the tumor cell origin. Genetic studies revealed that all cases of DSRCT harbor the t(11;22)(p13;q12) translocation, leading to a fusion of the N-terminal domain (NTD) of Ewing's sarcoma gene (*EWS*) to the C-terminal DNA binding domain of Wilms tumor suppressor gene, *WT1* [5], [6]. This unique chromosomal translocation provides the definitive molecular diagnosis of DSRCT and creates an aberrant transcription factor, EWS/WT1, which underlies the oncogenesis of DSRCT.

The *EWS* gene was first cloned from the Ewing's sarcoma chromosomal breakpoint, where the translocation generates a fusion between *EWS* and an ETS-family transcription factor gene, *FLI-1* [7]. *EWS* encodes a putative RNA binding protein, which together with TLS/FUS and TAFII68/TAF15 form the TET family of proteins with presumptive roles in transcription and splicing [8]. The NTD of EWS mediates potent transcriptional activation when fused to a heterologous DNA binding domain [9], while its C-terminal domain, which is lost in the translocation gene product, is involved in RNA recognition [10]. Recently, EWS was shown to be essential for meiosis, B-cell development and cellular senescence [11], as well as in mitosis [12].

The Wilms tumor suppressor gene *WT1* encodes a Kruppel-like transcription factor which is mutated in a subset of Wilms tumor, a childhood kidney cancer [13]. *WT1* encodes four Cys_2_His_2_ zinc-fingers in the C-terminus that mediate sequence specific DNA binding and the NTD containing both transcriptional activation and repression domains [14], [15]. *WT1* is subjected to two alternative splicing events, one of which involves the inclusion or exclusion of three amino acids, lysine, threonine and serine (KTS), between the zinc-fingers 3 and 4 [16]. The KTS insertion leads to a markedly decreased DNA binding affinity of WT1 [17]. In nearly all cases of DSRCT, only the last 3 exons of *WT1* encoding the last three zinc fingers are fused to the NTD of EWS, and the alternative KTS splicing of *WT1* is preserved [6]. As a result, *EWS/WT1* produces two isoforms: EWS/WT1(−KTS) and EWS/WT1(+KTS) that differs in the DNA binding affinity and specificity [18]. It was subsequently shown that only the EWS/WT1(−KTS) isoform, but not the EWS/WT1(+KTS), possesses the transforming activity in NIH3T3 cell-based assays [19]. Therefore, most efforts have focused on the identification of transcriptional targets of the EWS/WT1(−KTS) isoform.

To date, a small number of direct transcriptional targets of EWS/WT1(−KTS) have been identified (reviewed in [4]), which include *PDGF-A* (platelet-derived growth factor A) [20], *IGFR1* (insulin-like growth-factor receptor 1) [21], *IL2RB* (interleukin 2 receptor beta) [22], *BAIAP3* (BAI1-associated protein 3) [23], a potential regulator of growth-factor release, and *TALLA-*1 (T-cell acute lymphoblastic leukemia-associated antigen 1) [24], a gene encoding a tetraspanin-family protein. In contrast, there is only a single target gene identified for EWS/WT1(+KTS), which is *LRRC15* (leucine-rich repeat containing 15) [25], a gene implicated in cell invasion. The native WT1(±KTS) isoforms do not regulate any of these transcripts, indicating that the loss of zinc finger 1 and/or the presence of EWS transactivation domain (NTD) may confer differential target gene specificity to EWS/WT1. Another interesting observation is that all of the identified target genes are regulated specifically by either the EWS/WT1(−KTS) or the (+KTS), but not both, clearly demonstrating the differential target gene specificity of the two isoforms.

*ENT4* (equilibrative nucleoside transporter 4) is a member of the equilibrative nucleoside transporter (ENT) family (SLC29) and has recently been shown to be a pH-dependent adenosine transporter [26], [27]. Interestingly, ENT4 also accepts a number of biogenic amines as substrates and was alternatively named plasma membrane monoamine transporter (*PMAT*) [28]. In this study, we demonstrate that *ENT4* is transcriptionally activated by both isoforms of EWS/WT1 and is highly expressed in DSRCT, implicating *ENT4* as a potential therapeutic target.

## Results

### Identification of ENT4 as a target gene of EWS/WT1(−KTS) and EWS/WT1(+KTS)

**Figure 1:**
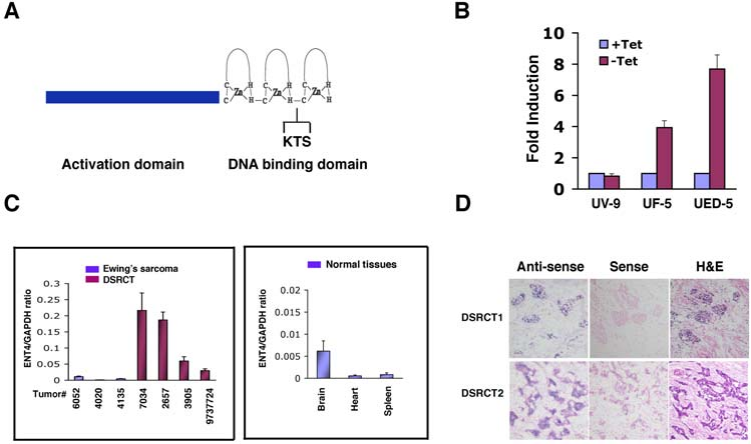
Identification of ENT4 as a target of EWS/WT1 in DSRCT. A. Schematic representation of EWS/WT1 chimeric protein. The activation domain of EWS and the three zinc fingers of WT1 (DNA biding domain) are indicated. KTS indicates the alternative KTS splicing. B. Induction of *ENT4* expression by both isoforms of EWS/WT1. Total RNA isolated from cells with Tet-repressible expression of EWS/WT1(−KTS) (UF-5), EWS/WT1(+KTS) (UED5) and empty vector (UV9) grown in the presence or absence of Tet for 12 hrs was analyzed by qRT-PCR using ENT4 TaqMan probe (Applied Biosystems). Fold induction represents the level of *ENT4* expression following the inducible expression of EWS/WT1 (−Tet) relative to the uninduced (+Tet). Data were analyzed by comparative Ct method using GAPDH as a control. C. Expression of *ENT4* in primary DSRCT and Ewing's sarcoma specimens, and in normal human tissues. Total RNAs isolated from primary tumors of DSRCT and Ewing's sarcoma specimens and from normal human brain, heart and spleen were analyzed for *ENT4* and *GAPDH* expression using TaqMan quantitative RT-PCR. Relative expression of *ENT4* is shown as the ratio of *ENT4* transcripts relative to *GAPDH* transcripts (note the scale difference in the left and the right panels). D. RNA *in situ* hybridization of *ENT4* in DSRCT. Two frozen DSRCT samples were sectioned and hybridized to *ENT4* riboprobes. *ENT4* expression is restricted to tumor cells (antisense probe). No staining was observed with a control (sense) probe. Tumor sections were also stained with hemotoxylin and eosin (H&E).

To identify the direct target genes of EWS/WT1(−KTS), we have previously performed expression profiling analysis with an inducible cell line expressing EWS/WT1(−KTS) [23]. This approach identified a number of EWS/WT1(−KTS) target genes which include several known genes and ESTs [4], [22], [23]. In the present study, we further characterized one of the EST transcripts (GenBank accession # R13346) which was induced by EWS/WT1(−KTS) expression with variable magnitudes in duplicate microarray experiments (21.0- and 3.1-fold induction). Sequence analysis revealed that the EST belongs to a recently identified novel monoamine transporter, *PMAT* [28], and independently identified as a new member of equilibrative nucleoside transporter, *ENT4*, with high-affinity for adenosine [29]. To validate this finding, we generated an independent tetracycline (Tet)-repressible EWS/WT1(−KTS) expressing cell line, UF5. A quantitative measurement of *ENT4* transcripts following the induction of EWS/WT1(−KTS) for 12 hrs in UF5 cells demonstrated a 4-fold increase in endogenous *ENT4* expression (Fig. 1B). The presence or absence of Tet in the control UV9 cells did not affect the level of *ENT4* expression. To determine whether the induction of *ENT4* was specific to the EWS/WT1(−KTS) isoform, we examined *ENT4* expression in UED5 cells expressing EWS/WT1(+KTS). Surprisingly, we observed a robust 7-fold induction of *ENT4* following the expression of EWS/WT1(+KTS), which is higher than the 4-fold induction observed with EWS/WT1(−KTS) expression (Fig. 1B). The difference in the magnitude of *ENT4* induction was not due to the difference in the expression levels of the two EWS/WT1 isoforms, since the −KTS was expressed at a higher level in UF5 cells than the +KTS in UED5 cells as demonstrated by SYBR Green quantitative RT-PCR (qRT-PCR) (Supplement Fig. S1). In addition, an inducible expression of native WT1, either the −KTS or +KTS, did not alter the level of endogenous *ENT4* expression (data not shown), demonstrating that *ENT4* is specifically activated by EWS/WT1. Thus, *ENT4* represents the first target gene which is transcriptionally activated by both isoforms of EWS/WT1.

### Tumor-specific expression of ENT4 in DSRCT

To establish *ENT4* as a physiological target of EWS/WT1, we next examined the expression of *ENT4* in primary DSRCT samples. Total RNAs from four DSRCT and three Ewing's sarcoma tumor specimens as well as normal human tissues were isolated and analyzed for *ENT4* expression by qRT-PCR. All four DSRCT specimens showed higher expression of *ENT4* than any of the Ewing's sarcoma samples (Fig. 1C, left panel) or the two tissues (brain and heart) where *ENT4* is reported to be highly expressed [26] (Fig. 1C, right panel). In fact, compared to the Ewing's sarcoma case (#6052) with the highest *ENT4* expression, DSRCT samples expressed at least 2- to 20-fold higher level of *ENT4* transcripts. Importantly, RNA *in-situ* analysis with two cases of DSRCT clearly revealed a tumor-specific *ENT4* expression with little or no expression in the surrounding stromal tissues (Fig. 1D). These results establish that *ENT4* is a physiological target of EWS/WT1 and is highly expressed by the tumor cells of DSRCT.

### ENT4 promoter is transactivated by both isoforms of EWS/WT1

**Figure 2:**
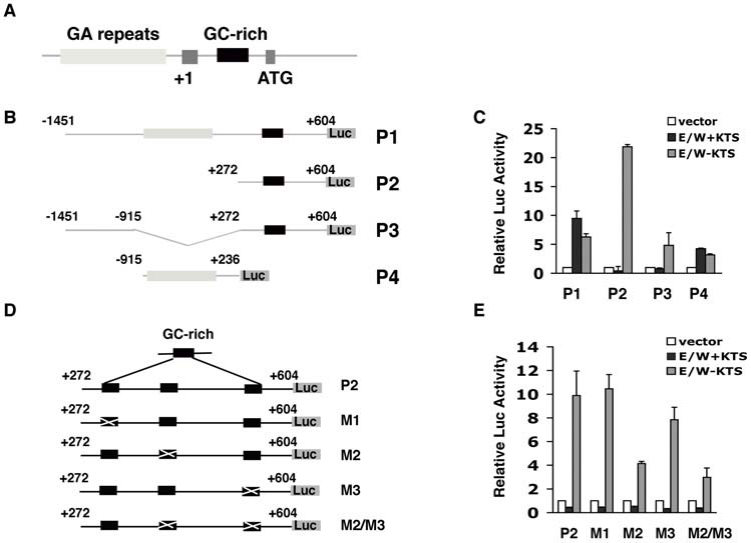
Identification of EWS-WT1(+KTS) and (−KTS) responsive elements in the human *ENT4* promoter. A. Schematic representation of *ENT4* gene. The dark grey box indicated by +1 represents the first non-coding exon and the transcription start site (+1). The second dark grey box marked by ATG represents exon 2 containing the starting ATG. The light grey box represents the GA-repeat sequence. The black box represents the GC-rich sequence. B. Schematics showing the luciferase reporter constructs (P1–P4) containing different fragments of the *ENT4* promoter. P3 promoter construct contains the deletion of GA-repeat sequences (−915 to +272). The numbers are relative to the transcription start (+1). C. Direct transcriptional activation of *ENT4* promoters by EWS/WT1. U2OS cells were co-transfected with either pcDNA3-EWS/WT1 (− or +KTS; 0.5 µg) or with pcDNA3 along with the promoter-reporter constructs (P1, P2, P3 or P4; 0.5 µg) and Renilla luciferase (0.1 µg) using FuGENE 6. Luciferase activity was measured at 48 hrs post-transfection and expressed as relative luciferase activity compared to the empty vector. Data represent the mean±S.D. from three independent experiments. D. Schematics showing the three putative EWS/WT1(−KTS) binding sites (black boxes) within the GC-rich region of the P2 promoter and the mutated derivatives, M1, M2, M3 and M2/M3 constructs (see Materials and Methods for detail). E. Identification of the EWS/WT1(−KTS) responsive element M2. Luciferase-reporter assay was performed as in (C) except with the promoter-reporter constructs shown in (D). Data represent the mean±S.D. from three independent experiments.

We next wished to determine whether EWS/WT1(±KTS) isoforms can directly and independently transactivate the promoter of *ENT4*. We amplified approximately 2-kb proximal promoter region of *ENT4*, including the first exon containing the 5′ UTR, (Fig. 2A) and inserted it into a promoterless pGL3-Basic luciferase plasmid (P1). Co-transfection of either EWS/WT1(−KTS) or EWS/WT1(+KTS) with the 2-kb promoter construct P1 resulted in a marked transcriptional activation of the *ENT4* promoter by both isoforms (Fig. 2C). Consistent with our previous results (Fig. 1B), EWS/WT1(+KTS) expression led to a higher activation of the *ENT4* promoter (10-fold) than EWS/WT1(−KTS) (6-fold). These results suggest that two isoforms can directly and independently transactivate *ENT4* expression.

### Identification of the −KTS and +KTS specific responsive elements in ENT4 promoter

EWS/WT1(−KTS) has been shown to bind to either a GC-rich sequence (5′-GXGGXGGXG-3′) [18] or a E-WRE sequence (5′-(G/C)(C/G)(G/C)TGGGGG-3′) [22]. On the other hand, EWS/WT1(+KTS) binds to a novel recognition sequence termed, E(KTS)RE, which has been defined as (5′-GGAGG(A/G)-3′) [25]. Inspection of the 2-kb human *ENT4* promoter sequences revealed three potential EWS/WT1(−KTS) responsive elements (GC-rich) located downstream of the transcriptional start site and a 760-bp stretch of GA-repeats, which contains more than 50 potential +KTS binding sites, E(KTS)RE motif, located upstream of the transcriptional start site (Fig. 2A). To test whether these sequences can function as the *cis*-regulatory elements, we generated various promoter deletion constructs (Fig. 2B). A deletion of 1700-bp region (−1450 to +270) upstream of the GC-rich sequences (promoter P2), which includes the GA-repeat sequences containing more than 50 potential E(KTS)RE elements, resulted in a complete loss of EWS/WT1(+KTS)-mediated transactivation (Fig. 2C). A smaller deletion encompassing mostly the GA-repeat sequences (promoter P3) also completely abrogated the +KTS-mediated transactivation of the *ENT4* promoter, demonstrating that the multiple E(KTS)RE sites in the GA-repeat sequences likely function as the EWS/WT1(+KTS) response element. In contrast, the P2 promoter (330-bp) containing mostly the GC-rich sequences was highly activated (21-fold) by EWS/WT1(−KTS), indicating that the GC-rich sequences likely mediate the transactivation by the −KTS isoform. EWS/WT1(−KTS) activated the P3 promoter to a similar level (5-fold) as the 2-kb P1 promoter (6-fold), indicating that the removal of negative elements in the 5′ upstream region (−1450 to −915) of the human *ENT4* promoter may be responsible for the augmented activation of the P2 promoter by EWS/WT1(−KTS). Deletion of the GC-rich sequences (P4) resulted in a reduced but not complete absence of transactivation by the −KTS, suggesting the presence of additional *cis*-elements for EWS/WT1(−KTS). Interestingly, removal of the GC-rich sequences (P4) also led to a reduction in the EWS/WT1(+KTS)-mediated transactivation, demonstrating that the GC-rich sequences are needed to achieve full transactivation by EWS/WT1(+KTS).

To precisely define the −KTS-specific response elements in the GC-rich region of the *ENT4* promoter, we introduced base substitutions within the three potential EWS/WT1(−KTS) binding sites of the P2 promoter (Fig. 2D). Mutation of the first potential binding site M1 at +370 (5′-GAGGGGGTC-3′ to GAAAAAATC-3′) had no effect on the EWS/WT1(−KTS)-mediated activation of the promoter (Fig. 2E). However, mutation of the second binding site M2 at +440 (antisense strand: 5′-GCGGGGGGG-3′ to 5′-GCGGAAAAA-3′) resulted in approximately 60% reduction in the activation of the P2 promoter by the −KTS isoform. Altering the third site M3 at +570 (5′-CTGGGGGCG-3′ to 5′-CTAAAAACG-3′) showed a slight decrease in the EWS/WT1(−KTS)-mediated transactivation, but it was not statistically significant. Consistent with this, mutations at both the M2 and M3 sites (the M2/M3 promoter) in the GC-rich region displayed a similar level of reduction in the reporter assay as the single M2 mutation (Fig. 2E), demonstrating that the M2, but not the M1 or the M3 sites, functions as the *cis*-regulatory element for EWS/WT1(−KTS). These observations also indicate the existence of other EWS/WT1(−KTS) responsive element(s) within the GC-rich region. EWS/WT1(+KTS) had no discernible activity on any of the base substituted promoters (Fig. 2E). We did not attempt to define the +KTS responsive element in the GA-repeat sequences due to the presence of more than 50 potential E(KTS)RE sites.

### EWS/WT1 is recruited to ENT4 promoter in vivo

**Figure 3:**
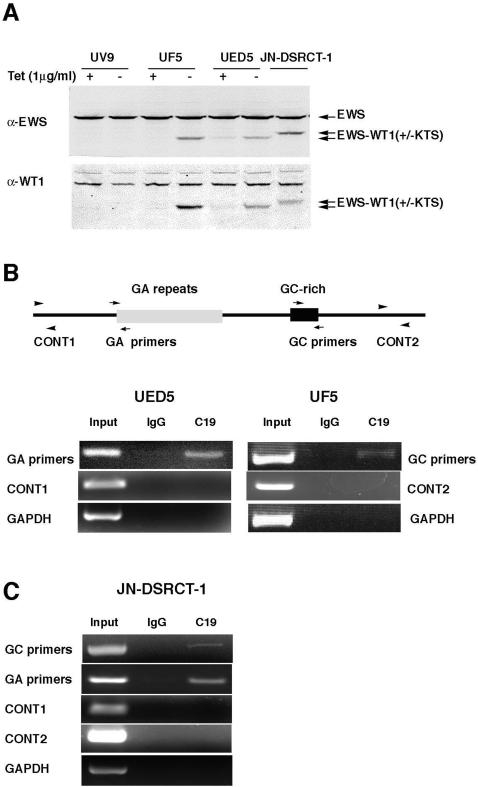
Direct *in vivo* recruitment of EWS/WT1(±KTS) to *ENT4* promoter. A. Western blot analysis of EWS/WT1 in the inducible cell lines (UF5 and UED5) and in the primary DSRCT cell line (JN-DSRCT-1), probed with anti-EWS antibody (upper panel) or with anti-WT1 antibody (lower panel, C-19, Santa Cruz). EWS/WT1 expression was induced (−Tet) for about 14 hrs in UF5 and UED5 cells. B. Chromatin immunoprecipitation (ChIP) analysis. Schematics of *ENT4* promoter and the locations of the primers (arrows) used to amplify the GA-repeat and the GC-rich regions are shown. EWS/WT1(+KTS) or (−KTS) expression was induced for 14 hrs in UED5 and UF5 cells, respectively, and crosslinked with 1% formaldehyde. Crosslinked chromatin immunoprecipitated with anti-WT1 antibody (C-19, Santa Cruz) or rabbit IgG control was used in PCR amplification with either primers in the GA-repeat (for UED5 cells) or the GC-rich (for UF5 cells) regions of the human *ENT4* promoter. Primers amplifying the control regions of the *ENT4* promoter (CONT1 and CONT2) and GAPDH promoter were used as negative controls. C. ChIP analysis in JN-DSRCT-1 cells. Formaldehyde-crosslinked chromatin from JN-DSRCT-1 cells was examined by ChIP analysis using the same primer sets as in (B).

We next performed chromatin immunoprecipitation (ChIP) assay to determine whether EWS/WT1 is recruited to the *ENT4* promoter *in vivo*. UF5 and UED5 cells were induced to express EWS/WT1(−KTS) and EWS/WT1(+KTS), respectively, and formaldehyde-crosslinked DNA was immunoprecipitated using an antibody that recognizes the C-terminal domain of WT1 (C-19) to enrich for the chromatin bound by EWS/WT1. The endogenous WT1 is not detectable in U2OS cells by western blotting (data not shown), and thus, the C-19 antibody should be specific for EWS/WT1. We designed primers flanking the GC-rich sequences to examine the EWS/WT1(−KTS) binding and a primer set near the 5′ region of the GA-repeat sequences for detecting the EWS/WT1(+KTS) recruitment (Fig. 3B). ChIP analysis of UF5 cells demonstrated that EWS/WT1(−KTS) was present near the GC-rich region of *ENT4* promoter as shown by the specific amplification of the GC-rich sequences with the chromatin immunoprecipitated with C-19 antibodies but not with the IgG-immunoprecipitated chromatin control (Fig. 3B, UF5 panel). Similarly, ChIP analysis of UED5 cells showed *in vivo* recruitment of EWS/WT1(+KTS) to the GA-repeat region of the *ENT4* promoter (Fig. 3B, UED5 panel). Recruitment of the EWS/WT1 isoforms to these regions of the *ENT4* promoter was specific since the control primers amplifying the regions either 1kb upstream from the GA-repeat region (CONT1) or 1kb downstream from the GC-rich region (CONT2) failed to amplify any product.

A DSRCT cell line, JN-DSRCT-1, is the only established cell line derived from a primary DSRCT specimen and naturally expresses both isoforms of EWS/WT1 [30]. The breakpoint of EWS/WT1 translocation in JN-DSRCT-1 cell line is different (intron 10 of *EWS*) from the prototypical EWS/WT1 translocation (intron 7 of *EWS*) expressed in UF5 and UED5 cells. Thus, JN-DSRCT-1 cells express slightly slower migrating isoforms of EWS/WT1 (Fig. 3A). Immunoblot analysis with the C-19 antibody further revealed that JN-DSRCT-1 cells do not express detectable levels of endogenous WT1. To determine whether naturally occurring EWS/WT1 is recruited to the *ENT4* promoter, we performed the ChIP analysis in JN-DSRCT-1 cells. Consistent with our previous ChIP results, EWS/WT1 was physically recruited near the two *cis*-elements in the *ENT4* promoter in JN-DSRCT-1 cells as demonstrated by specific PCR amplifications of both the GA-repeat and the GC-rich regions of the promoter with chromatin immunoprecipitated with antibodies to WT1 (C-19), but not with IgG (Fig. 3C). As expected, the control primers (CONT1, CONT2 and GAPDH) did not yield any PCR products. Since the antibody (C-19) recognizes both isoforms of EWS/WT1, we cannot demonstrate the recruitment of each isoform to the specific regions of the *ENT4* promoter in JN-DSRCT-1 cells. However, based on our previous results (Fig. 2C and 3B), it is likely that each isoform is recruited to the respective *cis*-regulatory elements of the *ENT4* promoter in JN-DSRCT-1 cells. Interestingly, we consistently observed a more robust amplification of the GA-repeat region than the GC-rich region in our ChIP assays (Fig. 3B and C), which may indicate the presence of more EWS/WT1 in the GA-repeat region (which contains more than 50 EWS/WT1(+KTS) binding sites). Together, these results demonstrate that EWS/WT1 is recruited to the proximal promoter region of *ENT4 in vivo*.

### ENT4 is highly and specifically expressed in JN-DSRCT cell line

**Figure 4:**
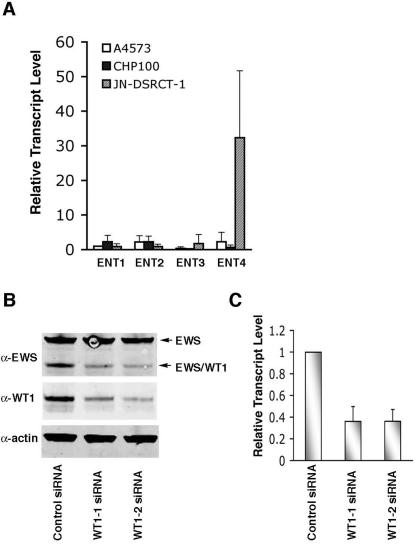
*ENT4* transcript is highly abundant in JN-DSRCT-1 cells and is regulated by EWS/WT1. A. Quantitative measurement of *ENT*-family transcripts. Total RNA isolated from JN-DSRCT-1 and two Ewing's sarcoma cells (A4537 and CHP100) was analyzed for relative transcript levels of *ENT*-family genes using Taqman probes for *ENT1*, *ENT2*, *ENT3*, and *ENT4*. *ENT1* transcript level from A4573 cells was arbitrarily set to 1 and used as a reference for quantification of other ENT-family transcripts in A4573 and other cells. Data were analyzed by comparative Ct method using GAPDH as a control. Data represent the mean±S.D. from three independent experiments. B. siRNA knockdown of EWS/WT1. JN-DSRCT-1 cells were transfected with either control, WT1-1 or WT1-2 siRNAs using Lipofectamine™ 2000, and at 48 hrs post-transfection, total cell lysates were analyzed by western blotting using anti-EWS, anti-WT1 and anti-actin (loading control) antibodies. C. Total RNA was isolated from the siRNA-transfected cells (as described in B) and the expression level of *ENT4* was determined by qRT-PCR as in (A). Data represent the mean±S.D. from three independent experiments.

We next sought to determine the expression level of *ENT4* in JN-DSRCT-1 cells. For comparison, we used two Ewing's sarcoma cell lines A4573 and CHP100, both of which express the EWS/FLI-1 translocation gene product. A quantitative measurement of the transcripts by qRT-PCR revealed that expression of *ENT4* was about 15- to 30-fold higher in JN-DSRCT-1 cells when compared to A4573 and CHP100 cells, respectively (Fig. 4A). Expression of other equilibrative nucleoside transporters (*ENT1-3*) was not enriched in JN-DSRCT-1 cells. Expression of *ENT4* was also at least 50-fold or higher in JN-DSRCT-1 cells than in three other human cancer cell lines examined, PC3, DU-145 and U2OS (Supplement Fig. S2). These results clearly demonstrate that *ENT4* is highly and specifically enriched in JN-DSRCT-1 cells, consistent with the highly abundant expression of *ENT4* in the primary DSRCT (Fig. 1C and D).

To further demonstrate that EWS/WT1 is directly regulating the *ENT4* expression, we used a small interfering RNA (siRNA) approach to deplete the level of EWS/WT1 in JN-DSRCT-1 cells. As shown in Fig. 4B, transfection of two different siRNAs directed against the 3′ UTR of *WT1* resulted in a marked reduction in the level of EWS/WT1 as compared to the control siRNA. Depletion of EWS/WT1 by two independent WT1-siRNAs concomitantly led to approximately 60% decrease in the *ENT4* transcripts (Fig. 4C), further supporting the direct transcriptional regulation of *ENT4* by EWS/WT1.

### Adenosine analog 2-CdA displays increased cytotoxicity in JN-DSRCT cells

**Figure 5:**
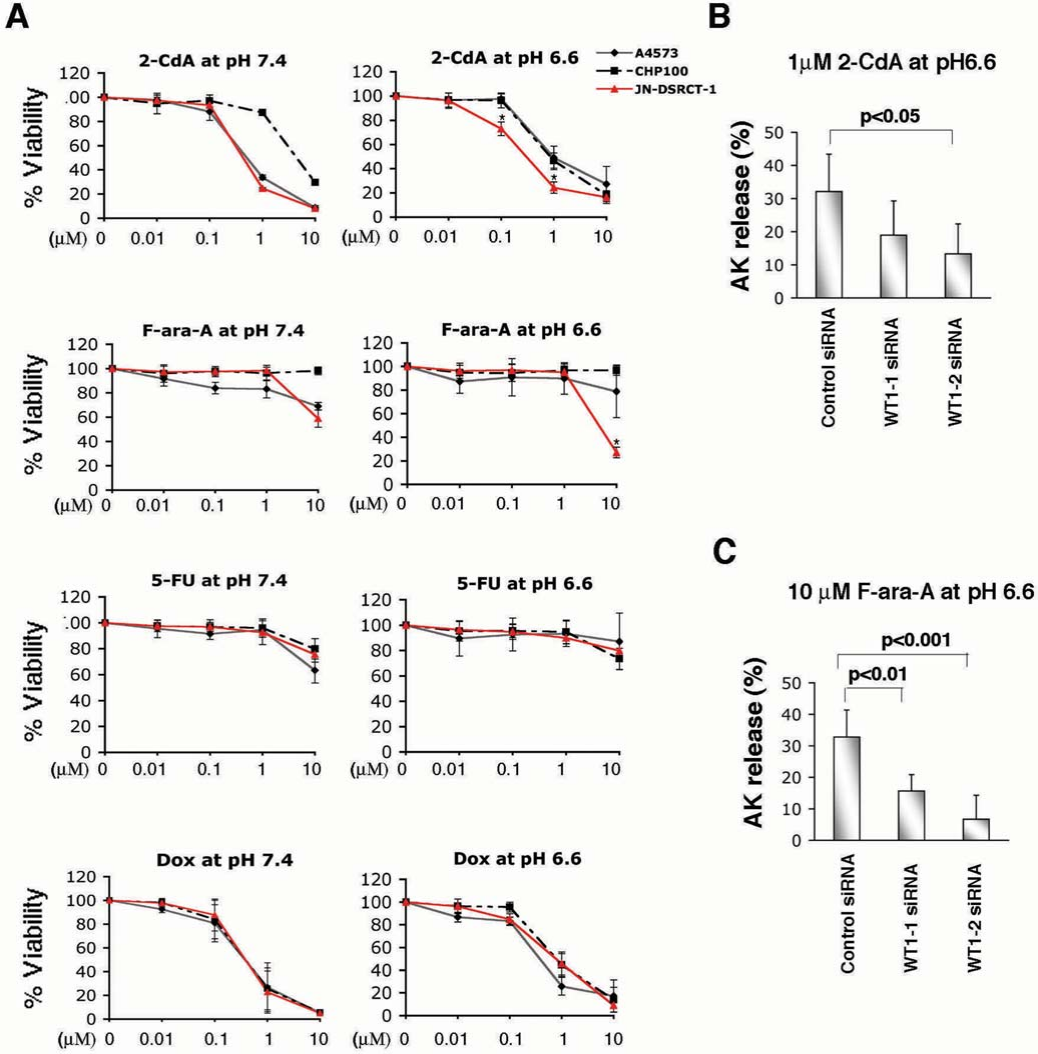
JN-DSRCT-1 cells display dose- and pH-dependent cytotoxicity to adenosine analogs. A. Cytotoxic assay. JN-DSRCT-1, A4573, and CHP100 cells were exposed to various concentrations (0.01–10 µM) of 2-CdA, F-ara-A, 5-FU or doxorubicin (Dox) for 72 hrs at pH 7.4 or pH 6.6. Cell viability was determined with Cell Counting Kit-8 (Dojindo Molecular Technologies). Data represent the mean±S.D. from three independent experiments performed in duplicate. *, *p* value < 0.01 was calculated by first performing one-way ANOVA analysis followed by Tukey's multiple comparison test. B and C. JN-DSRCT-1 cells were transfected with either control, WT1-1 or WT1-2 siRNAs using Lipofectamine™ 2000 for 24 hrs, and the cells were treated with either 1 µM of 2-CdA (B) or 10 µM of F-ara-A (C) at pH 6.6 for 48 hrs. The knockdown efficiency of ENT4 was confirmed by qRT-PCR (as in Fig. 4C) and cytotoxicity was assessed by Toxilight BioAssay Kit (see Materials and Methods). Data represent the mean±S.D. from at least three independent experiments performed in duplicate. *p* value was calculated by first performing one-way ANOVA analysis followed by Tukey's multiple comparison test.

Nucleoside transporters are thought to be important for the efficacy of nucleoside analogs used in chemotherapy such as gemcitabine, a pyrimidine nucleoside analog [31], [32]. Despite the initial classification of ENT4 as an adenosine transporter and a new member of equilibrative nucleoside transporter [29], ENT4 was shown to be a poor transporter of adenosine at neutral pH [28]. Recently, however, it was demonstrated that ENT4 exhibits a maximum adenosine transporter activity at pH 6.0 to 6.6 and is primarily localized at the plasma membrane [26], [27]. To examine whether we can exploit the high level of ENT4 expression and its adenosine transporter activity in DSRCT, we treated JN-DSRCT-1 and the control cells, A4573 and CHP100, with various nucleoside analogs: 2-chloro-2′-deoxyadenosine (2-CdA, also known as cladribine), 2-fluoroadenine-9-β-D-arabinofuranoside (F-ara-A, also known as fludarabine), or 5-fluorouracil (5-FU) as a pyrimidine analog control. For comparison, we also treated cells with doxorubicin, a highly cytotoxic chemotherapeutic compound used to treat DSRCT patients [33]. When cells were cultured at pH 6.6 and treated with a low dose (0.1 µM) of 2-CdA, JN-DSRCT-1 cells displayed a modest but significant increase in sensitivity to 2-CdA while the control A4573 and CHP100 cells remained fully viable under the same condition (Fig. 5A). Under the standard culture condition (pH 7.4), the same dose of 2-CdA (0.1 µM) had no cytotoxic effects on JN-DSRCT-1 cells (Fig. 5A). A higher dose of 2-CdA (1 µM) at pH 6.6 also led to a significant increase in cytotoxicity in JN-DSRCT-1 cells as compared to the control cells. At the highest dosage (10 µM), 2-CdA was highly cytotoxic to all cells examined. In contrast, different doses up to 1 µM of F-ara-A under pH 6.6 were not cytotoxic to any of the cell lines tested. However, 10 µM of F-ara-A at pH 6.6 resulted in a dramatic increase in cytotoxicity in JN-DSRCT-1 cells compared to the control cells (Fig. 5A). The same dose of F-ara-A (10 µM) at neutral pH 7.4 had only a marginal effect on JN-DSRCT-1 cell viability (see pH 7.4 panel). Increasing doses of 5-FU showed minimal cytotoxicity in all cells cultured at either pH conditions. Doxorubicin treatment at pH 6.6 resulted in a similar dose-dependent cytotoxicity as that observed under the neutral pH.

To examine whether reducing the *ENT4* transcript level by WT1-siRNA could decrease the cytotoxicity of 2-CdA and F-ara-A, JN-DSRCT-1 cells were transfected with control or WT1-siRNAs, followed by treatment with 1 µM of 2-CdA or 10 µM of F-ara-A at pH 6.6. Decreasing the level of ENT4 with WT1-2 siRNA resulted in a markedly reduced cytotoxicity to 2-CdA treatment (Fig. 5B). WT1-1 siRNA transfected cells also showed slightly reduced cytotoxicity to 2-CdA, but it did not reach statistical significance. By contrast, transfection of either WT1 siRNAs resulted in a significant reduction in F-ara-A (10 µM) mediated cytotoxicity at pH 6.6 (Fig. 5C). Collectively, these results are consistent with the cytotoxic effects of 2-CdA and F-ara-A being mediated, at least in part, through ENT4 in JN-DSRCT-1 cells.

## Discussion

DSRCT-specific EWS/WT1 translocation results in the production of two fusion proteins with distinct biological activities, which has not been observed in other EWS-related chromosomal translocations. Two EWS/WT1 isoforms possess different DNA binding specificity due to the alternative KTS splicing event [18]. In addition, EWS/WT1(+KTS) could potentially have a non-transcriptional role since WT1(+KTS) isoform has recently been demonstrated to bind to cellular mRNA [34] and participate in the control of translation [35]. Thus, the existence of two isoforms of EWS/WT1 in DSRCT raises the following interesting questions: what are the roles of each isoforms and are they both required for tumorigenesis? The latter question has been addressed by Kim et al [19] who demonstrated that, at least *in vitro*, only the EWS/WT1(−KTS) isoform possesses the transforming activity using the NIH3T3 cell-based transformation assays. However, it remains to be determined whether the −KTS isoform is sufficient for tumorigenesis *in vivo*.

To address the oncogenic role of EWS/WT1, most of the efforts have been focused on identifying the transcriptional target of the −KTS isoform due to its *in vitro* transforming property. Because of altered DNA binding properties of the −KTS and the +KTS isoforms, it is presumed that their target genes would be different. Consistent with this notion, nearly all of the EWS/WT1 target genes identified are transcriptionally regulated only by the EWS/WT1(−KTS) isoform [20]–[24]. In contrast, *LRRC15* is the only EWS/WT1(+KTS) target gene identified to date and is solely regulated by the +KTS [25]. Therefore, it was quite surprising to find that *ENT4* was transcriptionally activated by both isoforms of EWS/WT1.

The promoter analysis revealed that the transcriptional activation by two EWS/WT1 isoforms is mediated through different *cis*-regulatory elements within the *ENT4* promoter. The GA-repeat sequences, which contain more than 50 potential binding sites for EWS/WT1(+KTS), mediate transactivation by the +KTS, whereas the GC-rich sequences containing the responsive element, M2, functioned as the *cis*-regulatory elements for the −KTS-specific transactivation (Fig. 2). The existence of distinct *cis*-regulatory elements for each EWS/WT1 isoforms raises an intriguing possibility of synergistic transcriptional activation by the two isoforms on the *ENT4* promoter. However, coexpression of both isoforms did not result in a synergistic activation of the *ENT4* promoter (data not shown). Our findings further suggest that EWS/WT1(+KTS) may play a more prominent role in the activation of *ENT4* transcription than EWS/WT1(−KTS), based on the higher induction level of endogenous *ENT4* transcripts by the +KTS isoform (Fig. 1B), higher transactivation by the +KTS in our promoter-reporter analysis (Fig. 2C), and by our ChIP analysis in which we observed a more robust amplification of the GA-repeat region than the GC-rich region of *ENT4* promoter (Fig. 3C). A likely explanation for this is the presence of more than fifty EWS/WT1(+KTS) binding sites in the GA-repeat region. This may also help to explain the remarkably high level of *ENT4* expression observed in both primary DSRCT specimens and JN-DSRCT-1 cell line, as well as the lack of synergy between the two isoforms in our promoter assay (the +KTS isoform may simply saturate the promoter). A highly abundant expression of ENT4 may be useful as an additional diagnostic marker of DSRCT although more cases need to be evaluated. Our study further suggests that it may be possible to identify additional target genes of EWS/WT1(+KTS) by examining the proximal promoter regions for the GA-repeat sequences.

ENT4 is a member of the equilibrative nucleoside transporter (ENT) family (SLC29), which contains four isoforms [29]. ENT1 and ENT2 transport purine and pyrimidine nucleosides (*e.g.* uridine, adenosine, etc.) and their structural analogs. ENT3 is an intracellular nucleoside transporter, which may play a role in lysosomal transport of nucleosides [36]. ENT4 is unique in the ENT family as it is specific towards adenosine and adenosine analogs and does not significantly transport other nucleosides and their congeners [26], [28], [29]. The activity of ENT4 towards adenosine is low at pH 7.4, and recent reports have shown that ENT4 exhibits a maximum adenosine transport activity at pH 6.0 to 6.6 [26], [27]. The role of ENT4 in the genesis and progression of DSRCT is unknown, but it may have a role in supporting tumorigenesis by providing growth and survival advantages to the tumor cells in adapting to a tumor microenvironment. In general, the tumor microenvironement becomes hypoxic and acidic due to a limiting blood supply and increased acid production from the upregulation of glycolytic metabolic pathways by the tumor cells [37]. It has also been demonstrated that adenosine can stimulate angiogenesis under hypoxic conditions [38]. Thus, it is intriguing to speculate that DSRCT-specific expression of ENT4, a pH-dependent (acidic) adenosine transporter, might provide growth and survival advantages to the tumor cells (e.g. by stimulating angiogenesis) under hypoxic/acidic conditions, as well as increasing the nucleoside pool within the rapidly proliferating tumor cells.

The expression level and pattern of the ENTs are an important determinant for the therapeutic efficacy of anticancer nucleoside analogs since many nucleoside analogs used in cancer chemotherapy depend on nucleoside transporters to enter tumor cells to exert their cytotoxicity (reviewed in [39]). For example, the expression of ENT1 in leukemia and certain solid tumors is related to the clinical efficacy of a number of nucleoside analogs such as cytarabine (Ara-C) and gemcitabine [39]. In light of this, our results demonstrating the cytotoxic effects of 2-CdA and F-ara-A in JN-DSRCT-1 cells *in vitro* are highly intriguing. Adenosine analogs such as 2-CdA and F-ara-A are an effective chemotherapeutic agent for the treatment of hairy cell leukemia and chronic lymphocytic leukemia, respectively [40]–[42]. However, efficacy of these adenosine analogs in solid tumors has not been reported. Our finding of *ENT4* as a transcriptional target of EWS/WT1 highly expressed in DSRCT suggests that this transporter may represent an attractive pathway for targeting chemotherapeutic drugs into DSRCT.

## Materials and Methods

### Cell lines

UF5, U2OS-derived cell line with tetracycline-repressible EWS/WT1(−KTS) expression, was generated as described previously [20]. UED5 cells with tetracycline-repressive EWS-WT1 (+KTS) expression [25] and UV9 control cells [20] were previously described. UV9, UF5 and UED5 were maintained in Dulbecco's modified Eagle's medium (DMEM) with 10% fetal bovine serum (FBS) and 1 µg/ml tetracycline. A human DSRCT cell line, JN-DSRCT-1 (kindly provided by Dr. Nishio) [30] and Ewing's sarcoma cell lines, A4573 and CHP100 (kindly provided by Dr. Crystal Mackall, NCI), were maintained in 1∶1 mixture of DMEM and Ham's F-12 with 10% FBS.

### Quantitative RT-PCR analysis

Total RNA was isolated from cell lines or tumor tissues with RNA STAT-60 (Tel-Test Inc. Friendswood, TX) according to the manufacturer's instruction. Total RNAs from normal human heart, brain and spleen were purchased from Zyagen (San Diego, CA). Two microgram of total RNA was treated with 1U of DNase I (Invitrogen, Carlsbad, CA) and reverse transcribed with SuperScript First-Strand Synthesis System (Invitrogen). For quantification of *ENT4*, equal amount of first-strand cDNA was mixed with each assay-on–demand TaqMan probes and Taqman Universal Master Mix (Applied Biosystems, Foster City, CA), and amplified using ABI prism 7700 sequence detection system (Applied Biosystems). Data were analyzed by comparative Ct method using *GAPDH* as an endogenous control. For the quantification of *ENT4* in UV9, UF5 and UED5 cells, the level of *ENT4* transcripts (normalized to *GAPDH*) in the absence of tetracycline was expressed as fold differences to the level of *ENT4* in the presence of tetracycline (arbitrarily set to 1). For the quantification of *ENT4* in normal human tissues, primary DSRCT and Ewing's sarcoma specimens, the ratio of *ENT4* transcripts relative to the Glyceraldehyde-3-Phosphate Dehydrogenase (*GAPDH*) transcripts was calculated using the comparative Ct method. For the quantification of *EWS/WT1* expression in UF5 and UED5 cells, the relative expression was determined by using the Quantitect™ SYBR Green PCR kit (Qiagen, Valencia, CA). The primers used for this assay were: 5′-TGGATCCTACAGCCAAGCTCC-3′; 5′-TTGGTGTCTTTTGAGCTGGTCTG-3′. *GAPDH* was used for standardization. All experiments were done in duplicate and data from minimum of three independent experiments were analyzed.

### ENT4 promoter constructs and luciferase reporter assays

A 2.0 kb fragment containing the human *ENT4* promoter was obtained by PCR using human genomic DNA and inserted into the XhoI/Hind III sites of a promoterless firefly luciferase reporter pGL3-Basic plasmid (Promega, Madison, WI) to yield P1. The following primers were used: 5′ GCGAGATCTGGTGGAAAGTGAAGGAAGGGCC 3′; 5′ CCGTTCGAACTCATCAGCCGCAAAGTTGGCTC 3′. Three promoter-deletion constructs (P2, P3 and P4) were generated by digestion of P1 with restriction enzymes and ligation of promoter fragments. The mutant constructs (M1, M2, M3 and M2/M3) were generated using QuickChange Multi site-Directed Mutagenesis Kit (Stratagene, Cedar Creek, TX) using the following primers:

M1: sense, 5′-GCCCTCGCCATGGAAAAAATCGGCGCCACCGCC-3′ antisense, 5′-GGCGGTGGCGCCGATTTTTTCCATGGCGAGGGC-3′

M2: sense, 5′-CGAAGTTCCTCCCCGGATTTTTCCGCGCCACCCCATC-3′ antisense, 5′-GATGGGGTGGCGCGGAAAAATCCGGGGAGGAACTTCG-3′

M3: sense, 5′-GGGGTCTGTCCTTTGCCTAAAAACGCAGGTCCGCGAGC-3′ antisense, 5′-GCTCGCGGACCTGCGTTTTTAGGCAAAGGACAGACCCC-3′. The *ENT4* promoter-reporter constructs (0.5 µg) were cotransfected into U2OS cells using FuGENE 6 (Roche, Indianapolis, IN), along with 0.5 µg pcDNA3-EWS/WT1(−KTS), -EWS/WT1(+KTS), or empty vector and renilla luciferase reporter plasmid (0.1 µg) for normalization of transfection variance. Luciferase activity was assayed at 48 hrs post-transfection using the Dual-Luciferase Reporter kit (Promega). Data were calculated from minimum of three experiments performed in duplicate.

### Chromatin immunoprecipitation (ChIP) analysis

ChIP analysis was performed as previously described [43] using ChIP assay kit (Upstate, Lake Placid, NY). After induction of EWS/WT1(−KTS) and EWS/WT1(+KTS) expression by removal of tetracycline, formaldehyde was added to a final concentration of 1% and cells were incubated at room temperature for 10 min. Cells were lysed in SDS lysis buffer (1% SDS, 50 mM Tris pH 8.0, 10 mM EDTA) and DNA was sheared by sonication to achieve an average length of ∼500-bp. Samples were diluted 10-fold in ChIP Dilution Buffer and incubated overnight at 4°C with either rabbit polyclonal anti-WT1 (C-19, Santa Cruz, Santa Cruz, CA) or with rabbit IgG. Samples were incubated with Salmon Sperm DNA/Protein A Agarose-50% Slurry and bound complexes were eluted in elution buffer (1% SDS, 0.1 M NaHCO3). Samples were incubated 4 hr at 65°C to reverse crosslinking and proteins were digested with Proteinase K. Following purification of DNA fragments (Qiaquick PCR purification kit, Qiagen), *ENT4* promoter regions were amplified by PCR using primers (to detect EWS/WT1(−KTS) binding within the GC-rich region): 5′-ACCTGTCGGAGCCTTTGTCTG-3′; 5′-ATCAGCCGCAAAGTTGGCTCG-3′, which yields a 452-bp product, and with primers (to detect EWS/WT1(+KTS) binding within the GA-repeat region): 5′-TGCACAGCCCAGCTGGATGG-3′; 5′-CCCATTCTCCTACTCAGTCC-3′, which yields a 218-bp product. To control for specificity, the following primers were used: CONT1 primers: 5′-CTAGTTGGAGCAATGGACTG-3′; 5′-GCTGGCATACAGCAGGAGCC-3′ (which amplify ∼1 kb upstream region of the GA-repeats, yielding a 250-bp product); CONT2 primers: 5′-CGCAGTAGGCTTGGATGTGG-3′; 5′-AATGCTCCTCCTGCCACCTG-3′ (which amplify ∼1 kb downstream sequences of the GC-rich region, yielding a 400-bp product); and GAPDH promoter primers: 5′-TTTACGGGCGCACGTAGCTCA-3′; 5′-CACCTTCCCCATGGTGTCTGA-3′, which yields a 462-bp product.

### RNA in situ analysis

RNA in situ hybridization was performed as described [23]. SP6 and T7 flanked PCR templates were used to generate digoxigenin-labeled riboprobes (Roche Molecular Biochemicals). The following ENT4 sequences were used as probe: human *ENT4* (accession AK092242), nt 146–583 or nt 584–941. Primary DSRCT tumor samples were obtained from the Department of Pathology, Memorial Sloan Kettering Cancer Center. OTC-embedded samples were cut into 9 µm sections, fixed in 4% paraformaldehyde, digested with Proteinase K (4 µg/ml), treated with acetic anhydride, and dehydrated in increasing concentrations of ethanol. Sections were hybridized overnight with 1 ng/µl probe. Bound probe was detected using an alkaline phosphatase conjugated anti-digoxigenin antibody (Roche Molecular Biochemicals) followed by incubation with BM purple (Roche Molecular Biochemicals), an alkaline phosphatase substrate. Control sense riboprobes were tested for each tissue.

### siRNA knockdown analysis

JN-DSRCT-1 cells were transfected with two small interfering RNA (siRNA) against the 3′ UTR of *WT1* (siWT1-1 and siWT2-2, purchased from Sigma-Aldrich, St. Louis, MO; siRNA ID: SASI_Hs01_00130271 and SASI_Hs01_00130272, respectively) or control scrambled siRNA. At 48 hrs post-transfection, total RNA was isolated, reverse-transcribed and quantified by RT-PCR using the Taqman probes against *ENT4* as described above. The knockdown of EWS/WT1 was verified by western blotting using rabbit polyclonal anti-EWS (1∶1000 dilution, [11]), anti-WT1 (C-19) (1∶200 dilution, Santa Cruz Biotechnology) and anti-Actin (1∶1000 dilution, Sigma-Aldrich) antibodies. All blots were visualized by Odyssey infrared system (LI-COR Bioscience, Lincoln, Nebraska).

### In vitro cytotoxicity assays

Ewing's sarcoma cell lines, A4573 and CHP100, and the DSRCT cell line, JN-DSRCT-1, were seeded (5×10^4^ cells/well) in 24-well plates and allowed to adhere for 24 hrs prior to treatments with increasing doses (0.01 to 10 µM) of doxorubicin or various nucleoside analogs, 2-Chloro-2′-deoxyadenosine (2-CdA, also known as cladribine), 2-Fluoroadenine-9-β-D-arabinofuranoside (F-ara-A, also known as fludarabine), and 5-fluorouracil (5-FU). All chemicals were purchased from Sigma-Aldrich. For an acidic culture condition, DMEM-F12 medium (pH 7.4) was adjusted to pH 6.6 by the addition of non-essential amino acids (100×, Invitrogen). The cytotoxicity of various drugs were measured using the Cell Counting Kit–8 (Dojindo Molecular Technologies, Inc. Gaithersburg, MD), a variant of an MTT assay which measures the conversion of a substrate to a water soluble formazan salt by sepctrophotometric quantification at 450 nm [44]. Dose-response curve and standard deviation were determined from three independent experiments performed in duplicate. For siRNA knockdown experiments, 3×10^5^ of JN-DSRCT-1 cells were seeded in 12-well plate and transfected with 100 pmol of each siRNA using Lipofectamine™ 2000 (Invitrogen). Twenty-four hours post-transfection, cells were treated with either 1 µM of 2-CdA or 10 µM of F-ara-A at pH 6.6 for another 48 hrs. The knockdown efficiency of EWS/WT1 was confirmed by western blotting. The cytotoxicity was assessed by an independent method using ToxiLight BioAssay Kit (Lonza, Rockland, ME), which quantitatively measures the release of adenylate kinase (AK) from damaged cells [45]. The percent AK release was calculated using the following formula: (RLU_Treated_−RLU_Untreated_)/RLU_Untreated_×100, where RLU represents the relative luminescence unit. All *p* values were calculated using one-way ANOVA analysis followed by Tukey's multiple comparison test.

## Supporting Information

Figure S1 Quantitative RT-PCR analysis of EWS/WT1(+KTS) and EWS/WT1(−KTS) expression in UED5 and UF5 cells. Quantitative RT-PCR analysis of EWS/WT1(−KTS) and EWS/WT1(+KTS) expression in UF5 and UED5 cells. Total RNA was isolated from UF5 and UED5 cells grown in the presence or absence of tetracycline (Tet) for 14 hrs and expression of EWS/WT1 in the absence of Tet was quantified by SYBR Green PCR and expressed as relative to the level in the uninduced (+Tet). GAPDH was amplified as a reference normalization control.
(0.39 MB TIF)

Figure S2 Expression of ENT4 in human cancer cell lines. Quantative RT-PCR analysis of ENT4 in human cancer cell lines. Total RNA was isolated from two human prostate cancer cell lines, DU-145 and PC3, an osteosarcoma cell line U2OS, and the JN-DSRCT-1 cell line, and the expression level of ENT4 was quantified by quantitative RT-PCR using assay-on-demand TaqMan ENT4 and GAPDH probes (Applied Biosystems, Foster City, CA). Data were analyzed by comparative Ct method using Glyceraldehyde-3-Phosphate Dehydrogenase (GAPDH) as an endogenous control. The expression level of ENT4 in DU-145 cells was arbitrarily set to a reference value of 1 and used to compare the level of ENT4 expression in other cell lines.
(0.42 MB TIF)
