# Supplementary figures and images for: Adenosine Transporter ENT4 Is a Direct Target of EWS/WT1 Translocation Product and Is Highly Expressed in Desmoplastic Small Round Cell Tumor
Source: PLoS One. 2008 Jun 4;3(6):e2353. doi: 10.1371/journal.pone.0002353 (PMC2394657; doi:10.1371/journal.pone.0002353)

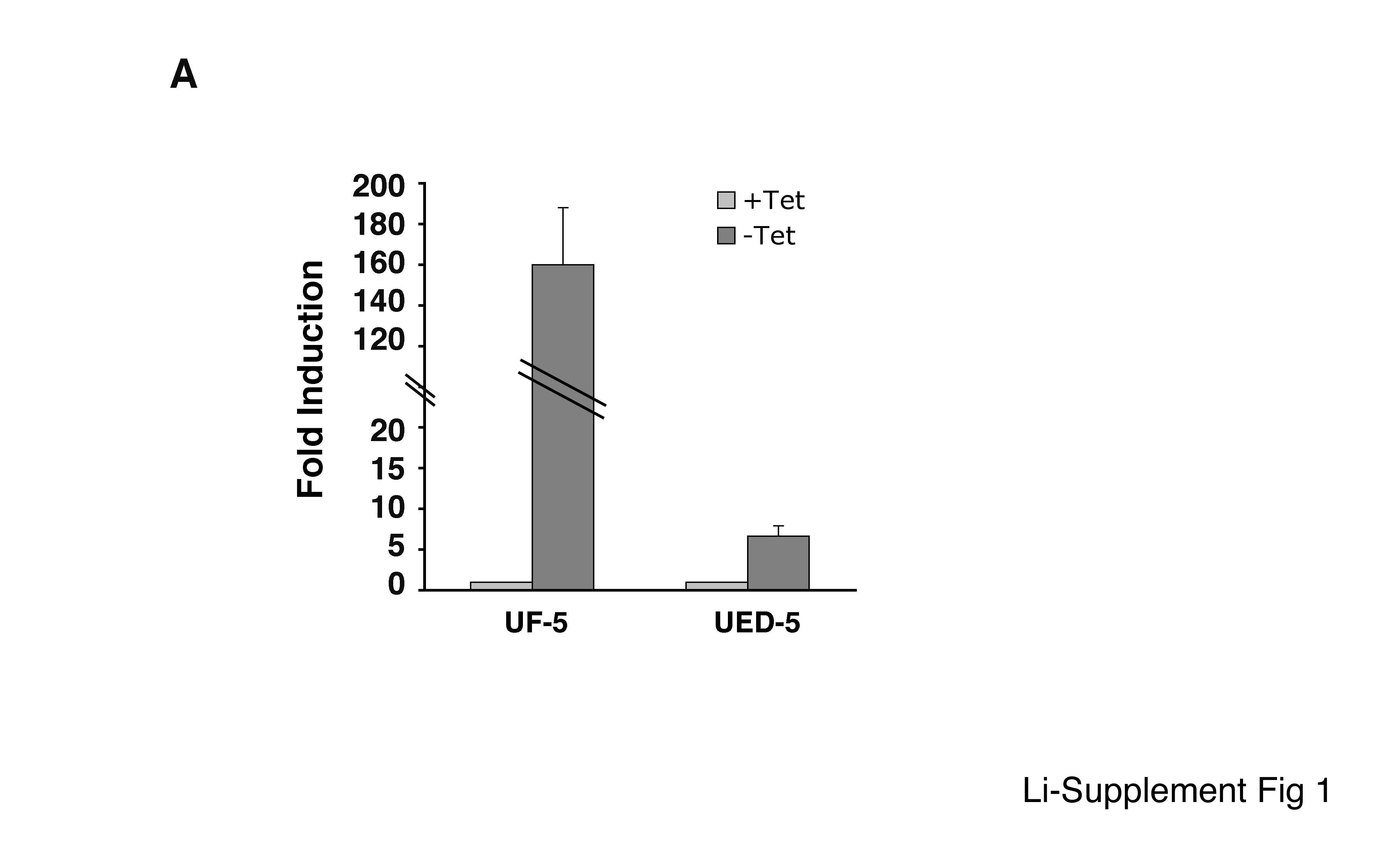

Supplement: Figure S1 — Quantitative RT-PCR analysis of EWS/WT1(+KTS) and EWS/WT1(−KTS) expression in UED5 and UF5 cells. Quantitative RT-PCR analysis of EWS/WT1(−KTS) and EWS/WT1(+KTS) expression in UF5 and UED5 cells. Total RNA was isolated from UF5 and UED5 cells grown in the presence or absence of tetracycline (Tet) for 14 hrs and expression of EWS/WT1 in the absence of Tet was quantified by SYBR Green PCR and expressed as relative to the level in the uninduced (+Tet). GAPDH was amplified as a reference normalization control. (0.39 MB TIF) [file pone.0002353.s001.tif]

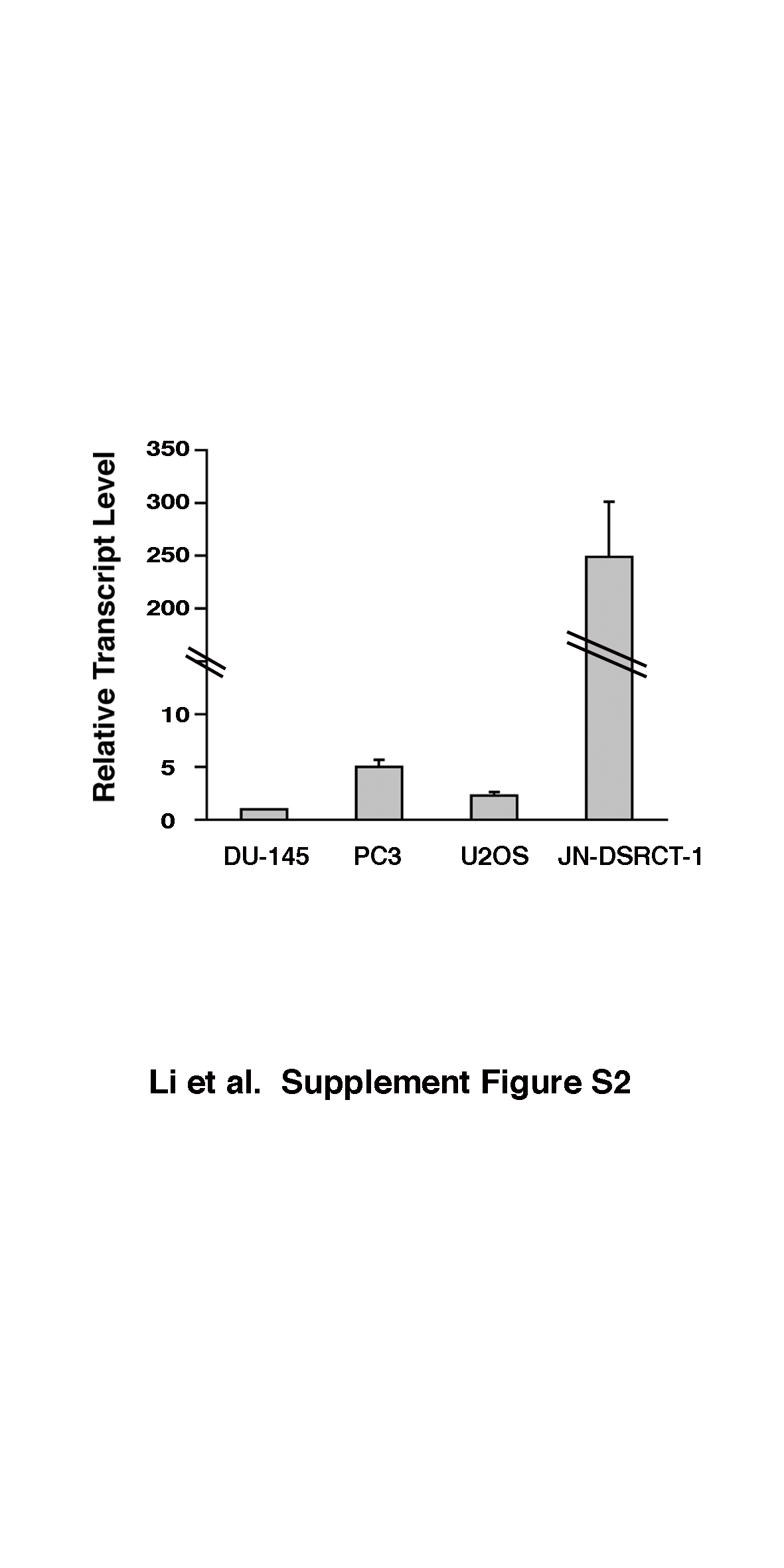

Supplement: Figure S2 — Expression of ENT4 in human cancer cell lines. Quantative RT-PCR analysis of ENT4 in human cancer cell lines. Total RNA was isolated from two human prostate cancer cell lines, DU-145 and PC3, an osteosarcoma cell line U2OS, and the JN-DSRCT-1 cell line, and the expression level of ENT4 was quantified by quantitative RT-PCR using assay-on-demand TaqMan ENT4 and GAPDH probes (Applied Biosystems, Foster City, CA). Data were analyzed by comparative Ct method using Glyceraldehyde-3-Phosphate Dehydrogenase (GAPDH) as an endogenous control. The expression level of ENT4 in DU-145 cells was arbitrarily set to a reference value of 1 and used to compare the level of ENT4 expression in other cell lines. (0.42 MB TIF) [file pone.0002353.s002.tif]
